# Three-dimensional morphological analysis of spermatogenesis in aged mouse testes

**DOI:** 10.1038/s41598-021-02443-4

**Published:** 2021-11-26

**Authors:** Taito Nakano, Hiroki Nakata, Suguru Kadomoto, Hiroaki Iwamoto, Hiroshi Yaegashi, Masashi Iijima, Shohei Kawaguchi, Takahiro Nohara, Kazuyoshi Shigehara, Kouji Izumi, Yoshifumi Kadono, Atsushi Mizokami

**Affiliations:** 1 Department of Integrative Cancer Therapy and Urology, Graduate School of Medical Sciences, Kanazawa University, Kanazawa, Japan; 2 Department of Histology and Cell Biology, Graduate School of Medical Sciences, Kanazawa University, 13-1 Takaramachi, Kanazawa, 920-8640 Japan

**Keywords:** Anatomy, Urology, Testis

## Abstract

Spermatogenesis, which is a continuous process from undifferentiated spermatogonia to spermatozoa in the seminiferous tubules, declines with age. To investigate changes in spermatogenesis with aging, we reconstructed the seminiferous tubules of 12 mice aged 12 to 30 months from serial sections and examined age-related and region-specific alterations in the seminiferous epithelium and spermatogenic waves in three dimensions. The basic structure of the seminiferous tubules, including the numbers of tubules, terminating points, branching points, and total tubule length, did not change with age. Age-related alterations in spermatogenesis, primarily assessed by the formation of vacuoles in Sertoli cells, were detected in the seminiferous tubules at 12 months. The proportion of altered tubule segments with impaired spermatogenesis further increased by 24 months, but remained unchanged thereafter. Altered tubule segments were preferentially distributed in tubule areas close to the rete testis and those in the center of the testis. Spermatogenic waves became shorter in length with age. These results provide a basis for examining the decline of spermatogenesis not only with aging, but also in male infertility.

## Introduction

Spermatogenesis is a continuous process from undifferentiated spermatogonia to spermatozoa in the seminiferous tubules^1–3^. Seminiferous tubules develop from the fetal testis cords and consist of a seminiferous epithelium, which is divided into 12 stages in mice according to the cell association pattern^4–9^. Adjacent stages are aligned along a seminiferous tubule, and the time required for a particular stage to reappear in the same area is called the cycle, while the space occupied by a series of adjacent stages, including all possible types, is called the wave^9,10^. Spermatogenesis is a lifelong process but its efficiency, such as daily sperm production, declines with age.

The decline in spermatogenesis with age is gradual and the complete cessation of reproductive capacity never occurs^11,12^. The impact of aging on semen parameters, such as sperm motility, sperm morphology, sperm DNA integrity, telomere length, and epigenetic factors, has been extensively examined^13,14^. However, the impact of aging on the testis structure remains unclear. The major morphological and functional alterations that occur in the testis with age include the formation of vacuoles in Sertoli cells, breakdown of the blood–testis barrier, thickening of the basement membrane, decrease in the number of Sertoli cells and spermatogonia, arrest of spermatocytes, formation of multinucleated spermatocytes, premature release of spermatids, and accumulation of lipofuscin granules in Leydig cells^15–23^. To the best of our knowledge, a three-dimensional (3D) analysis has not yet been performed on the structure of aged testis and it currently remains unclear whether the alterations described above occur preferentially at specific sites in the testis.

Our group recently reported the high-resolution 3D structure of all seminiferous tubules in animal testes using serial sections^24–28^. We detected region-specific morphological and functional differences within the testis. For example, we revealed that the sites at which spermatogenesis initially occurred in the postnatal mouse testis were preferentially distributed in areas of tubules close to the rete testis^25^, and that the extent of markedly impaired spermatogenesis was significantly greater in areas of tubules near the branching points in mice administered busulfan^26^. Furthermore, we performed an analysis of the waves, which is difficult using only 2D histological sections, and identified 76 waves with an average length of 16.9 mm in one adult mouse testis^25^. The biological meaning of the presence of waves may reside in the continuous and efficient production of spermatozoa without time intervals.

In the present study, to examine changes in spermatogenesis with aging, we reconstructed all of the seminiferous tubules of C57BL/6 mice aged 12 to 30 months from serial sections and performed a 3D analysis of age-related and region-specific alterations in the seminiferous epithelium and waves.

## Results

### 3D reconstruction of all seminiferous tubules

**Figure 1:**
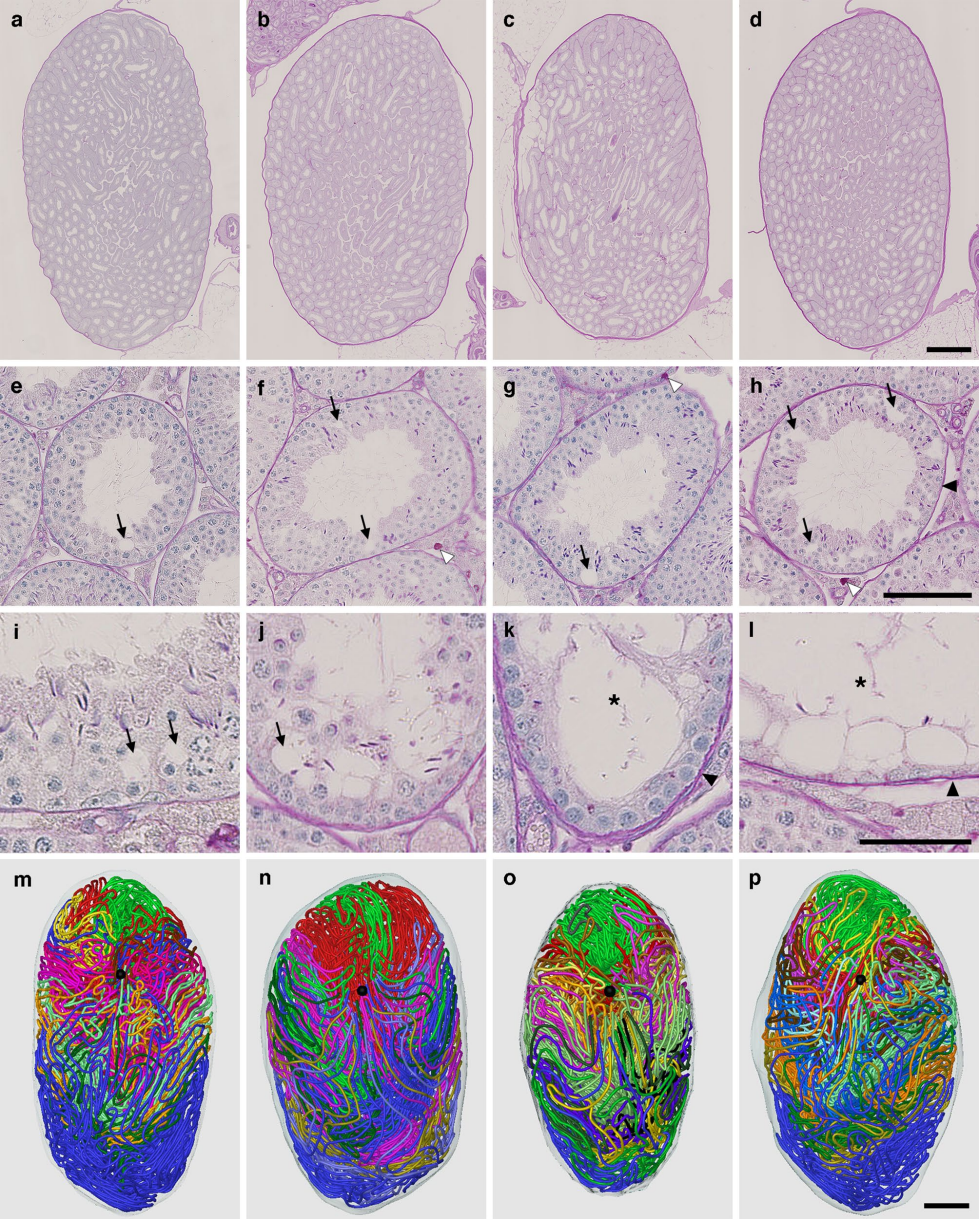
Reconstruction of all seminiferous tubules in representative testes at 12, 18, 24, and 30 months. (**a**–**l**) PAS-H-stained longitudinal sections of testes #2 (**a**,**e**,**i**), #5 (**b**,**f**,**j**), #7 (**c**,**g**,**k**), and #12 (**d**,**h**,**l**), which represent 12, 18, 24, and 30 months, respectively, are shown at low (**a**–**d**) and high magnifications (**e**–**l**). (**m**–**p**) The 3D core lines of all reconstructed seminiferous tubules marked with different colors are shown (**m**,**n**,**o**,**p** for testis #2, 5, 7, and 12, respectively) with the position of the rete testis (black sphere). Arrows, vacuoles in Sertoli cells; open arrowheads, PAS-positive materials in the interstitial compartment; closed arrowheads, thickening of the basement membrane; asterisks, tubules with severely impaired spermatogenesis. Scales, 1 mm (**a**–**d** and **m**–**p**), 100 µm (**e**–**h**), and 50 µm (**i**–**l**).

Twelve mice were divided into 4 groups of 3 mice each according to age: 12 (#1, 2, 3), 18 (#4, 5, 6), 24 (#7, 8, 9), and 30 (#10, 11, 12) months postpartum. All serial sections were stained with periodic acid-Schiff-hematoxylin (PAS-H) and all seminiferous tubules were reconstructed in 12 testes, one from each mouse. The results of 4 representative testes, one from each group, are shown in Fig. 1. As expected, progressive age-related histological changes, such as the formation of vacuoles in Sertoli cells to various degrees, strong PAS-positive materials in the interstitial compartment, dilation of the tubule lumen, and thickening of the basement membrane, were observed with age. However, no marked differences were noted in the 3D structure of the testis with age. Quantitative data on the seminiferous tubules are shown in Table 1. None of the values for the numbers of seminiferous tubules, terminating points, and branching points or the total length of seminiferous tubules per testis significantly differed among the 4 groups tested.

**Table 1:** Summary of reconstructed seminiferous tubules in aging mouse testes.

Testis	Months	Number				Total tubule length (mm)
		Total tubules	Terminating points^a^	Branching points	Blind ends	
#1	12	14	38	13	1	1523
#2	12	11	33	13	0	1758
#3	12	12	45	24	1	1539
#4	18	11	40	18	0	1778
#5	18	12	40	18	0	1879
#6	18	12	44	22	0	1877
#7	24	15	43	15	0	1466
#8	24	11	34	19	5	1335
#9	24	13	38	12	0	1614
#10	30	14	43	15	0	1309
#11	30	12	41	17	0	1919
#12	30	12	42	18	0	1783

^a^Connections with the rete testis.

### Analysis of age-related alterations in the seminiferous epithelium

**Figure 2:**
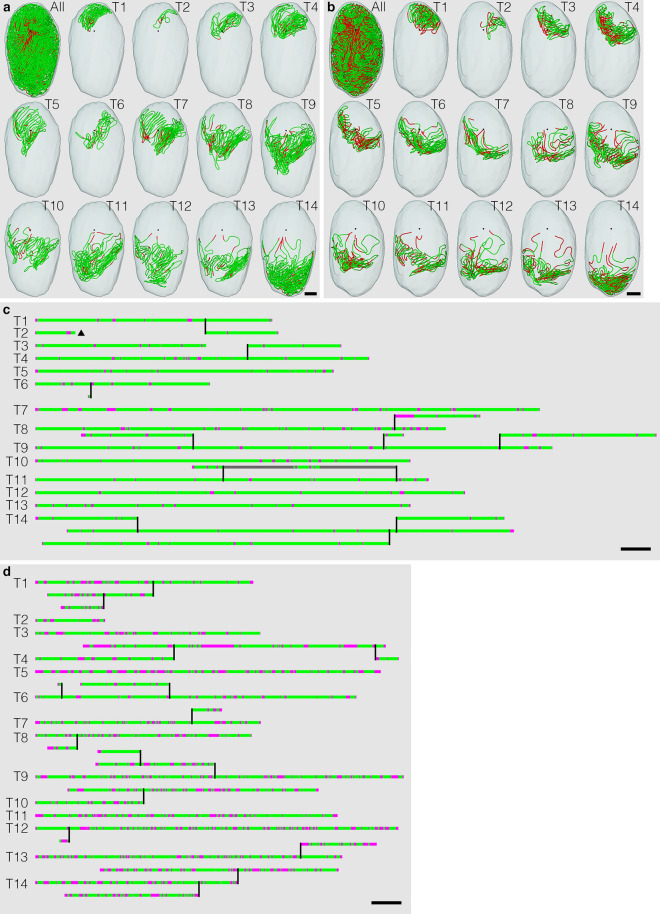
Classification of areas of seminiferous tubules in aged mice. Tubule areas were divided into two types with their epithelia: normal (green) and altered (red). Altered areas were identified with the presence of vacuoles in Sertoli cells and/or impaired spermatogenesis. (**a**,**b**) The core lines of all reconstructed seminiferous tubules in the testis #1 (12 months) and #10 (30 months), with areas belonging to the above two types marked with the corresponding colors, are shown in a vertical view with the position of the rete testis (black sphere). (**c**,**d**) The schemes of all individual seminiferous tubules in testes #1 and #10 are shown. The segments composed of tubule areas belonging to the two types are marked with the corresponding colors. Branching points are shown with vertical bars. Tubules are numbered from T1 to T14 in the order of their initial connection with the rete testis from the top toward the bottom in the testis. Except for one blind end (a black triangle) of T2 in testis #1, all free extremities of the tubules were connected to the rete testis in the terminal points without forming blind ends. Scales, 1 mm (**a**,**b**), 10 mm (**c**,**d**).

**Figure 3:**
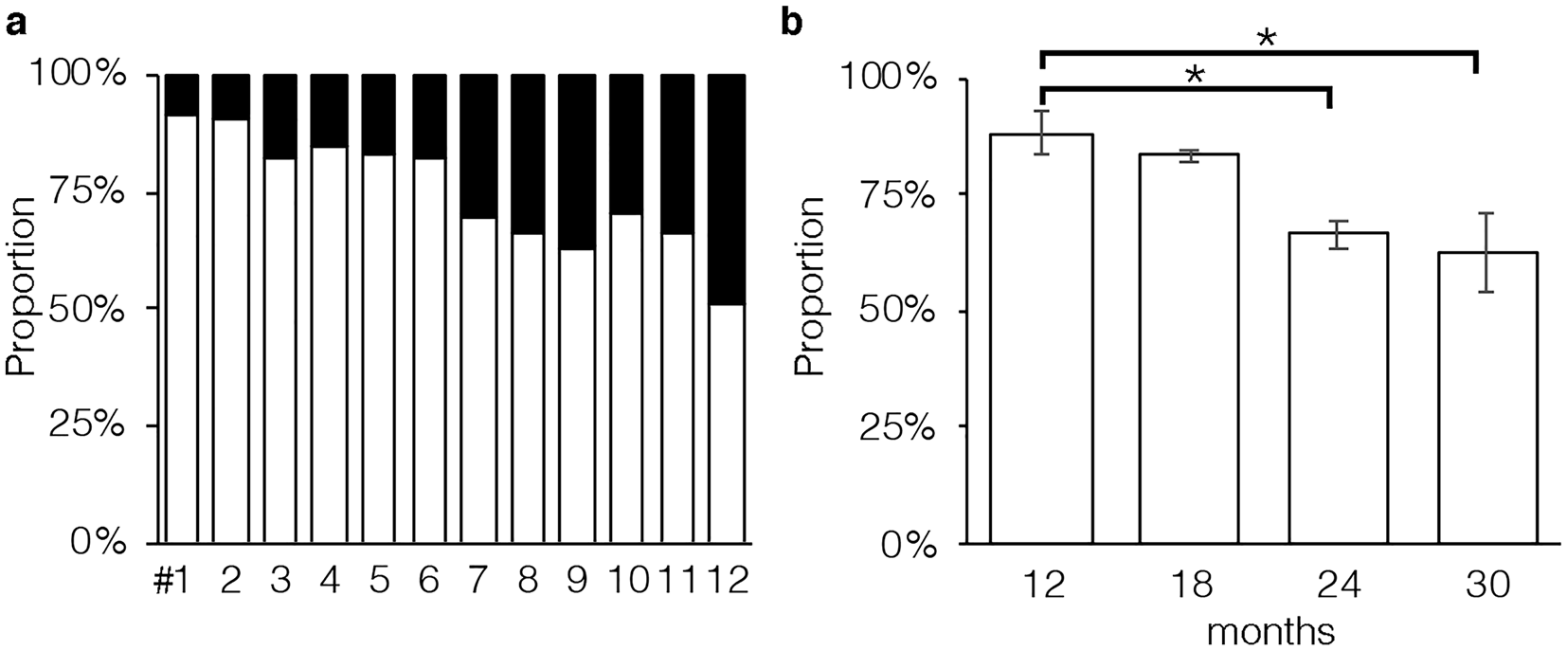
Proportions of tubule areas belonging to two types marked with corresponding colors in individual mice (**a**) and in groups of mice (12, 18, 24, and 30 months) (**b**). In (**b**), each value represents the mean ± SD of three samples. *Significantly different (P = 0.02).

The seminiferous epithelium consists of germ and Sertoli cells, with the latter exhibiting almost no proliferative or regenerative capacity in the adult testis. Therefore, to examine age-related alterations in the seminiferous epithelium, it is reasonable to assess the presence of vacuoles formed in Sertoli cells. All seminiferous tubule areas observed in each testis section were classified into two types according to the cell association pattern and the presence or absence of vacuoles formed in Sertoli cells: normal (green), normal spermatogenesis and the absence of vacuoles; altered (red), the presence of vacuoles (Fig. 1e–j) and/or impaired spermatogenesis (Fig. 1k,l). Based on this typing, the core lines of all seminiferous tubules in 12 testes were divided into segments of two colors (Fig. 2). The proportion of normal segments was 88 ± 4% at 12 months and did not change at 18 months. However, it significantly decreased (P = 0.02) at 24 months (66 ± 3%), but did not change further at 30 months (Fig. 3).

### Localization of sites of the altered seminiferous epithelium

**Figure 4:**
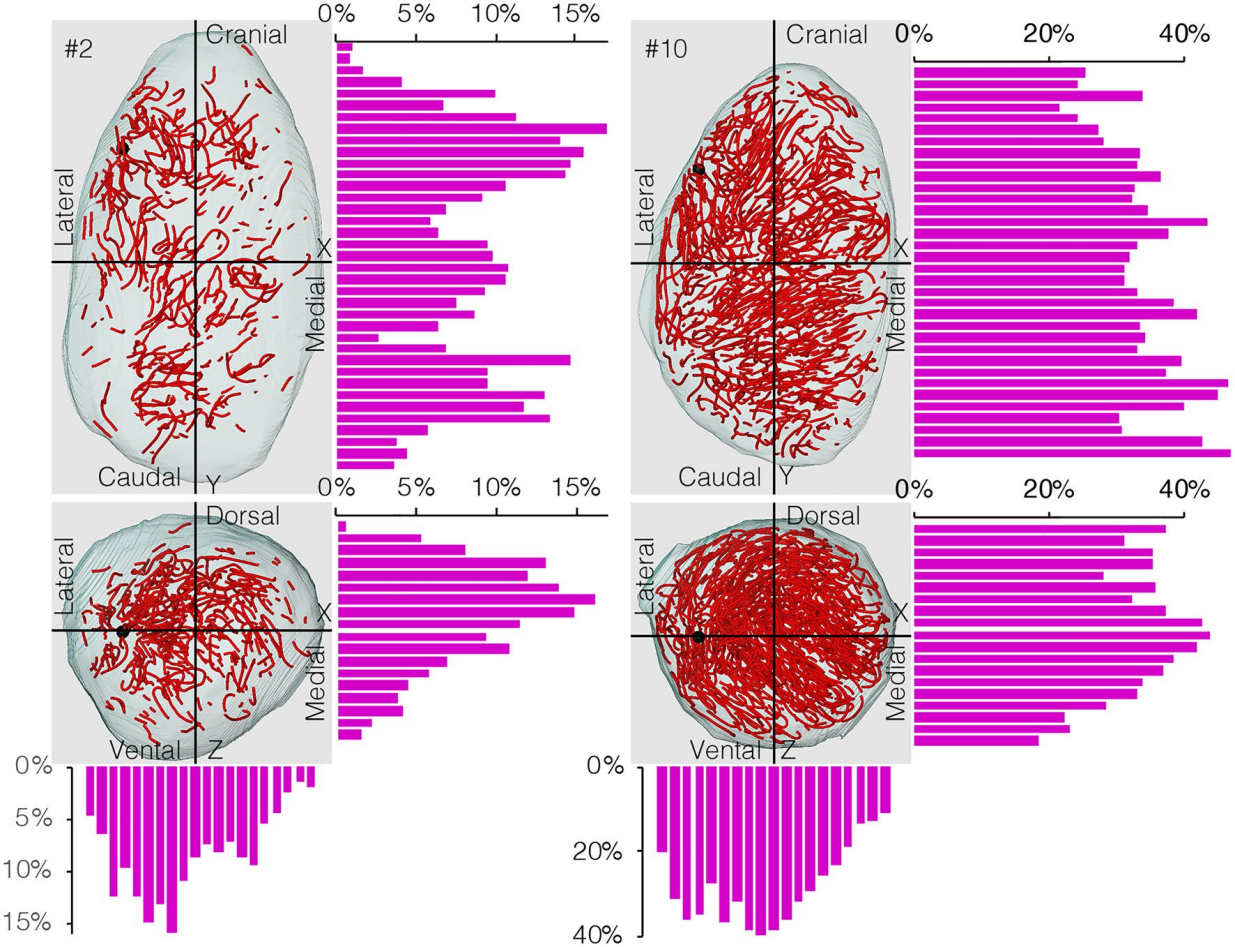
Proportions of altered tubule segments in different 3D positions in testes at 12 and 30 months. The proportions of the lengths of altered segments in testes #2 (12 months) and #10 (30 months) were measured in all tubule portions in 200-μm-thick serial slices of each testis parallel to the 3 planes formed by the x, y, and z axes. The altered segments and positions of the rete testis are shown in red and black spheres, respectively.

**Figure 5:**
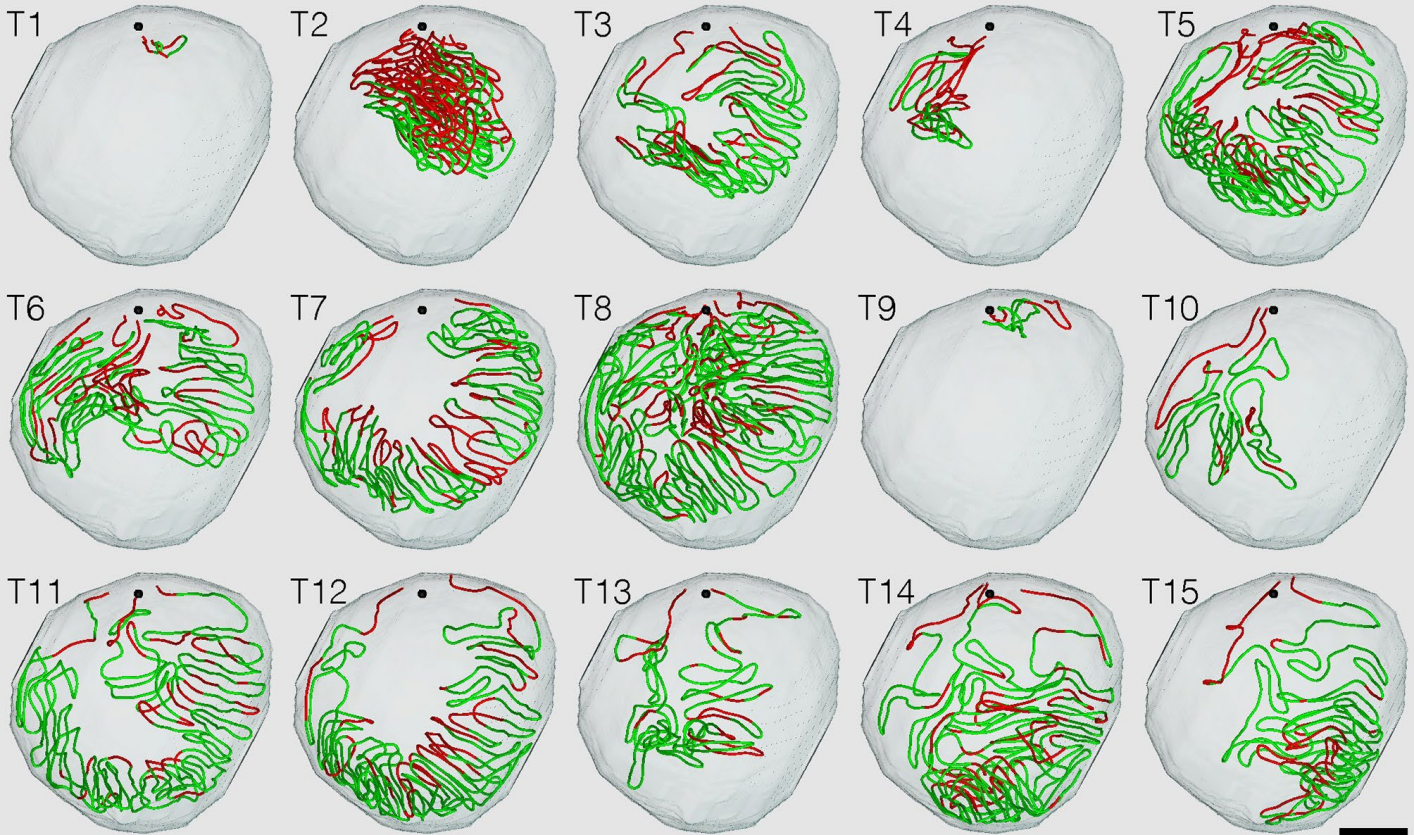
Distribution of normal and altered tubule segments at 24 months. The core lines of all reconstructed seminiferous tubules in testis #7 are shown in a horizontal view with the normal and altered segments shown in green and red, respectively, and the positions of the rete testis shown with black spheres. Tubules are numbered from T1 to T15 in the order of their connection with the rete testis from the top toward the bottom in the testis. Scale, 1 mm.

We examined whether the degree of alterations in the seminiferous epithelium differed among different 3D positions in the whole testis and in individual seminiferous tubules. A quantitative analysis of the 3D distribution of altered segments was performed in 12 testes, one from each mouse with the rete testis situated at the upper left corner, as in Fig. 4. The proportions of the lengths of altered segments against those of all seminiferous tubule portions present in 200-μm-thick serial slices parallel to 3 planes formed by the x, y, and z axes were shown in bars. In #2 testis (representing 12 months), the proportion of altered segments was larger in the central regions than in the peripheral regions of the testis close to the tunica albuginea along the x, y, and z axes. However, in #10 testis (representing 30 months), this regional difference was smaller along the z axis and absent along the y axis. Moreover, in the #2 and #10 testes, the proportion of altered segments was larger in the lateral half of the testis closer to the rete testis than in the medial half of the testis divided by the y–z plane. Similar results to those of #2 and #10 testes were obtained in the 5 other testes aged 12–18 months and 24–30 months, respectively. As shown in Fig. 5 for #7 testis (representing 24 months), when observing reconstructed seminiferous tubules from the direction of the rete testis, most of the areas connected with the rete testis, i.e., the terminal points, belonged to altered segments (98% of all terminal points in all 12 mice). This was also confirmed in the schemes of individual seminiferous tubules divided into the two types of segments, as shown in Fig. 2. Additionally, there were fewer altered segments in the tubule areas corresponding to the cranial hairpin turns in contact with the tunica albuginea than in those corresponding to the caudal turns occupying the central areas of the testis. The results shown in Fig. 5 are consistent with those in Fig. 4.

### Analysis of age-related alterations in spermatogenic waves

**Figure 6:**
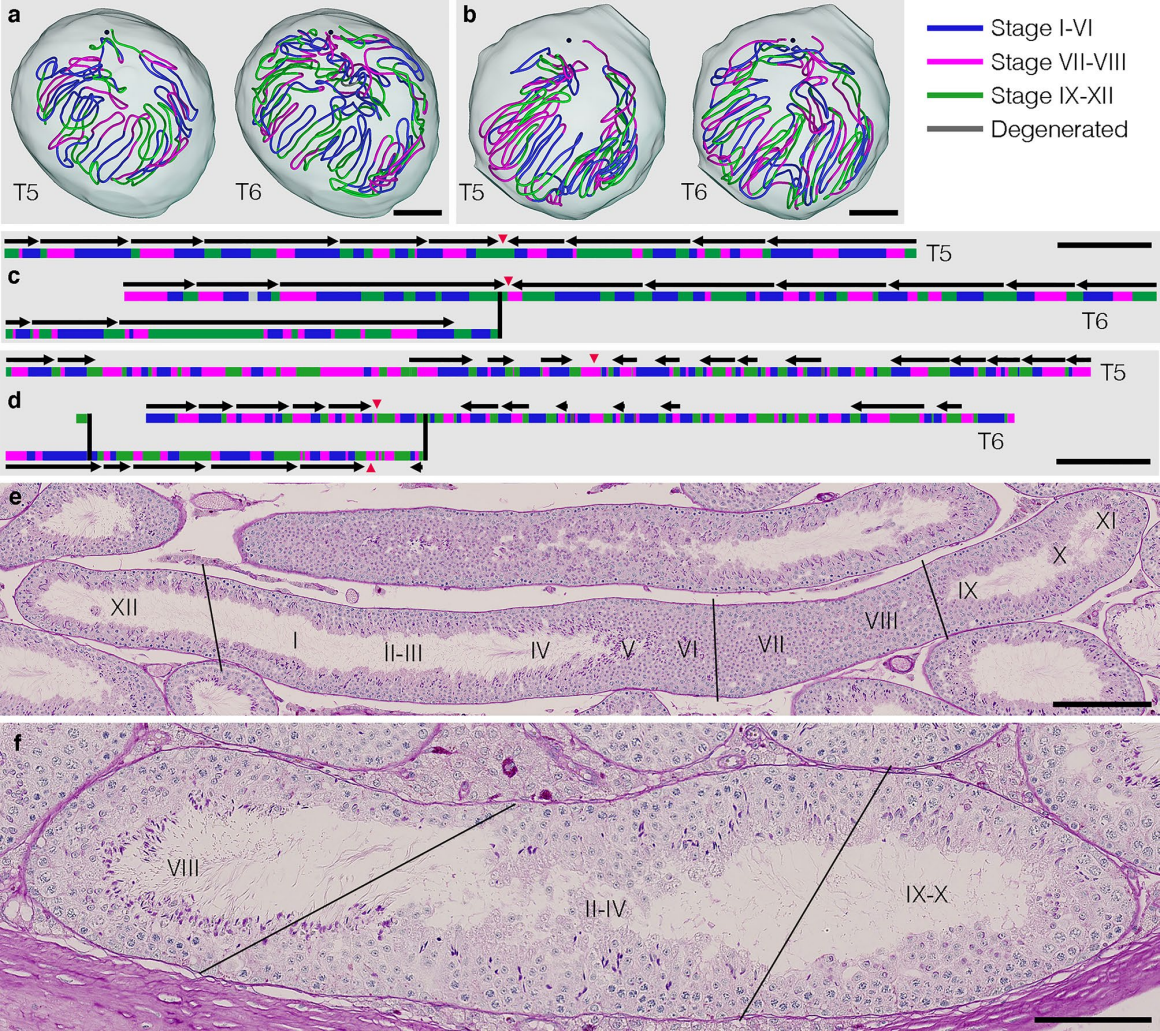
Distribution of spermatogenic stages and waves at 12 (**a**,**c**) and 30 (**b**,**d**) months. The core lines of reconstructed T5 and T6 tubules in testes #2 (12 months) and #10 (30 months) are shown in a horizontal view with the 3 + 1 different stage groups being marked in different colors and the positions of the rete testis being shown with black spheres (**a**,**b**). The different stage groups are as follows: stages I–VI (blue), stages VII–VIII (pink), stages IX–XII (green), and degenerated (gray). The schemes of the T5 and T6 tubules in testes #2 and #10 are shown in (**c**) and (**d**), respectively. The segments belonging to the 3 + 1 stage groups are marked with the corresponding colors. Horizontal arrows indicate the scopes and directions of waves. PAS-H-stained sections of testis #11 (30 months), with changes in stages with short intervals (**e**) and the irregular appearance of non-adjacent stages (**f**), are shown. The roman numerals in (**e**,**f**) represent stages I–XII. Scales, 1 mm (**a**,**b**), 10 mm (**c**,**d**), 200 µm (**e**), and 100 µm (**f**).

To analyze age-related alterations in spermatogenic waves, the stages of all seminiferous tubules in each section were assessed in #2 and #10 testes representing 12 and 30 months, respectively. All 12 stages were identified in PAS-H-stained sections based on the cell association pattern and the shape of acrosomes stained with PAS. The stages of the seminiferous epithelium are generally aligned in a descending manner from the rete testis side. However, this basic pattern of the arrangement of stages is often interrupted by local irregularities in the order of stage numbers. Furthermore, individual stages are too short in length for a 3D analysis. Therefore, to avoid complexity, we divided the seminiferous epithelium into 3 groups, i.e., stages I–VI, VII–VIII, and IX–XII. In addition, there were degenerated segments, the stages of which were unclear because of severely impaired spermatogenesis, as shown in Fig. 1k,l. The wave is defined as the space occupied by a series of adjacent stages, containing all possible types, for which there are 12 in mice. The core lines of all seminiferous tubules in testes #2 and #10 were divided into segments of different colors representing the 3 groups. The results obtained on 2 representative seminiferous tubules from both testes are shown in Fig. 6a–d. Quantitative data of the stage groups and waves are shown in Table 2. We detected 142 and 196 waves in #2 and #10 testes, respectively. The average lengths of these waves were 11.3 ± 6.0 and 3.7 ± 2.1 mm (P < 0.001), with the longest waves being 36.3 and 11.1 mm and the shortest being 3.0 and 0.8 mm, respectively. These results revealed that waves appeared to increase in number and significantly decrease in length with age. Regarding stages, all 3 stage groups appeared to increase in number and significantly decrease in length (P < 0.001 for stages I–VI, VII–VIII, and IX–XII), which is consistent with the results obtained on waves, whereas the proportions of the lengths of the 3 stage groups did not appear to change with age. In PAS-H-stained sections at 30 months, we often observed sites at which the stage changed with short intervals (Fig. 6e). The direction of waves, which leave from the rete testis side for the middle of each tubule with a lower stage number located distal to the preceding higher stage number, was reversed at the site of reversal. The relative number of sites of reversal in #10 testis against #2 testis (15:12) was similar to that of waves (196:142) (Table 2), indicating that the direction of the wave is not disordered with age. However, in addition to sites of reversal, we often found sites at which non-adjacent stages were aligned and unable to be included in waves at 30 months (Fig. 6f).

**Table 2:** Summary of stages and waves in seminiferous tubules.

Testis	Months	Stage groups								Wave		
		I–VI		VII–VIII		IX–XII		Degenerated^a^				
		Number of segments	Average length (mm)	Number of segments	Average length (mm)	Number of segments	Average length (mm)	Number of segments	Average length (mm)	Number	Average length (mm)	Site of reversal^b^
#2	12	324	2.4 ± 1.7^†^	309	1.5 ± 1.2^†^	312	1.7 ± 1.4^†^	13	1.0 ± 1.0	142	11.3 ± 6.0^†^	12
#10	30	584	1.1 ± 0.7	606	0.8 ± 0.7	594	0.8 ± 0.7	26	1.1 ± 1.7	196	3.7 ± 2.1	15

^a^The portion with the absence of spermatogenesis.

^b^The site at which the direction of waves is reversed.

^†^Mean ± S.D., significantly different between #2 and #10 (P < 0.001).

## Discussion

The present study revealed that the extent of age-related alterations in the seminiferous epithelium represented by vacuoles formed in Sertoli cells, which were already found at 12 months, increased significantly by 24 months, but remained unchanged thereafter. Even at 30 months, more than 60% of areas in the seminiferous epithelium retained a normal morphology. Since the average lifespan of C57BL/6 mice is approximately 28 months^29^, the present results are consistent with previous finding showing that the decline in spermatogenesis with age is gradual and the complete cessation of reproductive capacity never occurs^11,12^. Additionally, aging did not alter the basic structure of the seminiferous tubules, including the numbers of tubules, terminating points, branching points, and total tubule length.

The present study clearly demonstrated that the altered epithelium was preferentially distributed in areas close to the rete testis and the center of the testis. The area involving the junction between the rete testis and a seminiferous tubule forms a short special structure called the transition region (TR)^30^. This structure is different from other seminiferous tubules: TR consists of modified Sertoli cells that proliferate, shows distinct functions, and is potentially vulnerable to inflammation. Moreover, immune cells are abundant in TR^31^. Therefore, chronic inflammation may promote region-specific alterations in the areas of seminiferous epithelium close to the rete testis with age. On the other hand, excess heat may promote region-specific alterations in the seminiferous epithelium in the center of the testis with age. Excess heat on the testis causes germ cell apoptosis and Sertoli cell dysfunction^32,33^. Temperature may increase more frequently in the central regions of the testis than in the surface regions beneath the tunica albuginea, because the latter are more efficiently cooled with open air than the former. The difference in the proportion of the altered epithelium between the surface and central regions of the testis became smaller at 30 months than at 12 months, suggesting that alterations in the seminiferous epithelium due to excess heat proceeds toward the surface regions with age. The surface regions of the testis mainly consist of cranial hairpin turns of seminiferous tubules, which had higher proportions of the normal epithelium than the caudal hairpin turns occupying the central regions of the testis. Therefore, the zig-zag convoluted structure of seminiferous tubules may be a mechanism by which the tubule portions close to the tunica albuginea are distributed evenly along the lengths of all seminiferous tubules and may maintain the integrity of individual tubules.

The wave is defined as a series of adjacent tubule segments that contains all 12 stages in mice. A tubule portion occupied by an epithelium belonging to one of the 12 stages designated with roman numerals forms a segment. When a seminiferous tubule is traced from the rete testis, each segment is followed by a segment with the next smallest stage number. This observation suggests that the rete testis or other segments affect the stage of the adjacent segments^34^. The preservation of the wave is important for the maintenance of male fertility because the preservation of asynchronous germ cell differentiation constantly supplies numerous spermatozoa throughout life. The present study revealed that the segments become shorter and, as a result, the waves become shorter in length and larger in number with age. This phenomenon may be interpreted as the weakening of intercellular communications, including one with intercellular bridges, preventing the formation of large segments with age. This appears to be supported by the observation of sites at which non-adjacent stages were aligned such that the definition of waves is impossible at 30 months.

The region-specific alterations in spermatogenesis in aged mice, as revealed in the present study, are different from those in mice with busulfan-induced spermatogenic disorder^26^. By applying the present method, it may be possible to determine the specific patterns of the distribution of altered tubule portions in various types of spermatogenic disorders, including human infertility. The current limitations in applying the present study to the clinical purposes include the difficulty in obtaining the specimens from infertile human males, and the time and labor required for the 3D reconstruction that prevents the analysis of human testes with extremely large sizes.

## Methods

### Animals

Male C57BL/6 mice were purchased from Nippon SLC, Inc. (Hamamatsu, Japan), reared under standard 12-h light/12-h dark laboratory conditions with free access to standard food and water, and used at different ages: 12, 18, 24, and 30 months postpartum. All animal experiments complied with the ARRIVE guidelines. All procedures were approved by the Animal Care Committee of Kanazawa University (approval number: AP-173897) and were performed in accordance with relevant guidelines and regulations.

### Tissue preparation and PAS-H staining

Animals were sacrificed by cervical dislocation. The testis and epididymis were dissected out en bloc, fixed in Bouin’s solution overnight, dehydrated in a graded ethanol series, and embedded in paraffin. Five-µm-thick serial sections with intervals of 50 µm were cut using a microtome and mounted on glass slides. Sections were treated with PAS-H to stain the basement membrane of seminiferous tubules, as previously described^35^. Sections were digitized using a whole-slide scanner (Nanozoomer 2.0-HT C9600-13; Hamamatsu Photonics, Hamamatsu, Japan) with a 20-fold objective lens, and the resulting digital images of the sections were visualized with viewer software (NDP.view2 U12388-01; Hamamatsu Photonics).

### Reconstruction processing

The 3D reconstruction was performed as previously described^25–28^ with slight modifications. Briefly, extraction of the basement membrane stained in red–purple with PAS-H, which represents the outline of each seminiferous tubule, was performed in digital images using ImageJ software (NIH; Bethesda, MD, USA; http://imagej.nih.gov/ij/) and/or Adobe Photoshop 2020 software (Adobe Systems, Inc., Mountain View, CA, USA). After extraction, images were converted into gray scale in the JPEG format with Adobe Photoshop 2020 software at a resolution of 2724 nm pixel^−1^. Using Amira 6.3.0 software (Thermo Fisher Scientific, Waltham, MA, USA), serial images were automatically aligned followed by manual adjustments, and the inside of the outlines of a selected tubule was filled with a particular color using threshold processing and traced from section to section. This procedure, called segmentation, was repeatedly applied to all seminiferous tubules with different colors, and they were then 3D reconstructed. The core lines of individual seminiferous tubules were drawn using the same software. The whole testis was also 3D reconstructed by filling the inside of their outlines by threshold processing. The position of the rete testis was defined as the mean coordinate of the connections of all reconstructed seminiferous tubules with the rete testis and shown with a black sphere.

### Assessment of age-related alterations in the seminiferous epithelium

The appearance of vacuoles in the seminiferous epithelium is the first visible age-related change at the light microscopic level^12^. The seminiferous epithelia of all seminiferous tubules in each section were assessed by histological observations using NDP.view2 and classified into two types according to the cell association pattern and the presence or absence of vacuoles: a normal epithelium with normal spermatogenesis and the absence of vacuoles; an altered (abnormal) epithelium with the presence of vacuoles and/or impaired spermatogenesis. According to this typing, the core lines of all reconstructed seminiferous tubules were divided into segments of two colors. To analyze age-related alterations in spermatogenic waves, seminiferous epithelia were classified into 12 stages according to the germ cell association pattern by histological observations using NDP.view2 and the core lines of all reconstructed seminiferous tubules in 2 testes were divided into segments of different colors representing the 3 groups, i.e., stages I–VI, VII–VIII, and IX–XII. Furthermore, a wave was identified as a tubule segment containing successive stages of all 12 types. Waves were successively identified from the terminal points of tubules with the rete testis and marked on the core lines.

### Statistical analysis

Data were presented as the mean ± standard deviation (SD). Comparisons between two values were performed with the Mann–Whitney U test, and comparisons among multiple values were performed with the Kruskal–Wallis test followed by post hoc Dunn's test, with differences with a P-value of less than 0.05 being acknowledged as significant. Statistical analysis was undertaken using GraphPad Prism version 7.0e (GraphPad Software, San Diego, CA, USA).

## Data Availability

The original contributions presented in the present study are included in the article, and further inquiries may be directed to the corresponding author.
